# The inverted-U model of employee happiness: examining overdose happiness in context of personal characteristics, job-relationship dependency, benign stress, and various theories

**DOI:** 10.3389/fpsyg.2024.1285070

**Published:** 2024-05-21

**Authors:** Serap Kalfaoğlu

**Affiliations:** Selçuk University, Konya, Türkiye

**Keywords:** IUMEH, employee happiness, job-relationship dependency, benign stress, flow theory

## Abstract

In recent years, the management literature has begun to deal with individual and organizational results after happiness rather than the pursuit of happiness in business life and finally reaching happiness. After the fact that everything in an overdose is harmful, it has become the subject of even more research with paradoxical results that happiness that evokes positive emotions is not as innocent as it seems. In this study, which aims to reveal the harmful effects of overdose of employee happiness, the reasons for the manager’s fear - or anxiety - about the happiness of his employees are interpreted. The Inverted-U Model of Employee Happiness (IUMEH) has been developed and individual work outputs have been evaluated in three areas that (1) support happiness, (2) reflect balanced happiness, and (3) turn negative with an overdose of happiness intoxication. It has been suggested that IUMEH, which is thought to contribute to the literature as it is the first descriptive model to emerge, should be supported by applied studies, and it has been reminded that the curvilinear aspect of the model may include differences in terms of culture, type and characteristics of job, private, public or non-profit enterprises, generations of managers and the level of managers (front-line, middle level and senior level etc.).

## 1 Introduction

The questions of how to be happy or whether being happy should be seen as a goal or a way of life come to the fore in almost the majority of life. The pursuit of happiness is among the basic needs for most of them. Sometimes people miss the moment for that pursuit of happiness. As soon as he thinks that he has achieved full happiness, how he will reflect happiness and how he will behave becomes blurred (Türk et al., 2017). In their study on this subject, Mauss et al. (2011) concluded that the value given to being happy decreases when people find happiness, and therefore they do not feel very happy. In addition to the pursuit of happiness and finally reaching happiness, researches (Yaprak et al., 2018; Limone et al., 2020; An and Suh, 2023; Cheng and Jiang, 2023) have also been interested in post-happiness outcomes (Stončikaitė, 2019). The strange thing is that happiness is not as innocent as it seems.

In the past, countries with higher Gross Domestic Product (GDP) and *per capita* income were also among the highest in happiness rankings (Pincheira and Garcés, 2018). However, studies conducted over time have revealed that improvements in areas such as health, education or economy in people’s lives do not necessarily lead to an improvement in their happiness levels (Diener, 2000). In fact, the happiest countries have started to rank first in suicide rates (Atasoy and Ertürk, 2014). With the increase of researchers with similar views, studies have moved to the organizational arena and ideas and researches have begun to be carried out, accompanied by observations, on the reasons that will cause employees to be affected positively or negatively (Paschoal et al., 2010). However, despite the increasing interest in the factors that create organizational happiness in general, little information has been produced on the antecedents and foundations that explain organizational happiness (Salas-Vallina et al., 2020; Ataíde et al., 2023). Moreover, studies examining the effects of the dose of happiness on employees are limited. It also receives limited attention in management research.

The inspiration for this study is the work of Gruber and colleagues in 2011. Researchers have evaluated the dark sides of happiness in terms of intensity, timing, seeking, and types. *Intensity* refers to the level of happiness in which extreme or strength is felt strongly. Risky behaviors may be exhibited during this period of being away from reality. *Timing* tells you that happiness can have negative consequences when experienced in any situation. It is tiring as it is not possible to achieve constant happiness. While interpreting emotions and feelings from cognitive, physiological and social perspectives, it emphasizes interpersonal communication about the reactions received from the environment, how they react to them and how this social interaction will result. On the other hand, fear, which is one of the emotions that can be felt during happiness, increases the assessment of the situation and attention to any danger, threat or risk. The fear experienced is reflected in the decision-making behavior, and while expressing fear, there may be anxiety in the environment, which may present a sign for them to be helped (Sugay, 2019). In a way, fear is helpful and helps guide people around. *Pursuit of happiness* carries a paradox in it. The more people chase happiness, the harder it can be to achieve it. Finally, as *types of happiness*, Gruber et al. (2011) defined happiness that disrupts social functioning and is incompatible with cultural values. The first describes the supportive emotions that create the destructive effect in social relationships. Arrogance can be given as an example. Arrogant people weaken their relationships over time. The subject of culture, on the other hand, refers to the diversity in the interpretation of emotions with its distinctive feature for societies.

In this study, not cherophobia, which is called the fear of one’s own happiness, but the fear of someone else’s happiness, − the manager’s fear or anxiety about the happiness of the employee in particular - is discussed. As the antithesis of employee happiness, the manager’s feeling of fear and fear-based thoughts were evaluated. It has been shown that the reason for the manager’s fear of the happiness of his employees is his personality traits, interdependence, benign stress, as well as the idea that his employees will not be able to focus on work after an overdose of happiness, they will experience an intoxication of happiness, and that every good and fun event will be bad after all. As a result of the investigations, inspired by Yerkes and Dodson (1908), the “Inverted-U Model of Employee Happiness (IUMEH)” was developed in this study. In the related study, it is aimed to reveal the individual and organizational harmful aspects of overdose employee happiness in business life, and it is thought that a significant contribution has been made to the literature, especially by presenting a model for the managers to interpret the operation correctly and to improve the self-awareness of the employees.

This study is a descriptive research with literature review. Literature review is the summary, synthesis and analysis of information about the research problem (Balcı, 2013). Survey models, on the other hand, are research approaches that aim to describe a past or present situation as it is (Çevik, 2020). In this model, the investigated event, individual or object is tried to be defined within its own conditions without any intervention (Karasar, 2011). The literature review was supported by theories and also the effect of personal observations was taken into account. In the study, first of all, it was desired to make sense of happiness and employee happiness. Although it is relative and incommensurable, definitions and findings from various aspects are included and a framework is presented for the readers. The effect of the overdose of happiness on the employee’s work life was discussed in the light of research and the manager-employee relations were interpreted from the perspective of personality traits and addiction. Finally, a visual of what was explained throughout the study was presented with the IUMEH. This study considered a selective range of evidence, while also incorporating studies with varying outcomes. Through a systematic process of the revealed IUMEH, it was possible to observe similarities and differences among the included studies. The study offers a unique perspective on employee happiness and its significance in the business world, rather than simply repeating previous research. Considering the immaturity of the subject under investigation, it is believed that the literature review has successfully undergone the design, implementation, data synthesis, and reporting stages. It is hoped that the model will contribute to the management literature.

## 2 The problem of relativity and inability to measure happiness and employee happiness

From time immemorial philosophers, religious leaders, artists, poets, writers and psychologists have gone out to explore happiness, dedicating most of their time to understanding it. But happiness is a very complex subject. It is neither easy to understand, manage nor measure. The ancient Greeks referred to the distinction between instant and long-term happiness while expressing happiness as *hedonia* and *eudaimonia*. They compared instant pleasure with long-term satisfaction.

Today, there is no clear definition of happiness that is accepted by everyone. For Samuel Johnson, happiness is momentary, it does not last. For Jean-Jacque Rousseau, it is drifting aimlessly in a boat and feeling like a god (Spicer and Cederström, 2015). In the literature, the concept of happiness is often associated with various positive emotions (Olumekor et al., 2022; Rizzato et al., 2022). Definitions of happiness have changed over the years. For some, it describes an inner experience spent with emotions such as happiness, pleasantness, contentment, pleasure, joy, abundance, self-actualization, and personal development (Andrews and Withey, 1976; Ryff, 1989). For some, it is an emotion-oriented assessment of one’s well-being (Diener, 1984). For some, it explains the pleasure from life that appears emotionally in individuals (Moyano and Ramos, 2007). While each of these perspectives may seem logical, no single perspective is sufficient to summarize the full meaning of happiness (Chamorro-Premuzic, 2020). In common usage, it is seen as synonymous with subjective well-being (Baluku et al., 2021, 2024). Therefore, happiness is relative and it is not clear exactly what it is (Misra and Srivastava, 2023). However, since there is an opportunity to examine the happiness literature from different perspectives, the following personal definition can be made: Happiness is the joy felt when it is achieved, the warmth felt in the soul, the feeling of intoxication that postpones future dreams, the illusion of short-term success and a choice that inhibits the emotional regulation mechanism. Happiness is a joy because it occurs when the desired phenomenon is achieved. Happiness is the feeling of warmth in the soul because the inner reckoning is sincere. Happiness delays the pursuit of future dreams because today’s complacency prevents the individual from new adventures for a long time. Happiness is the illusion of success because it is momentary, not an all-encompassing phenomenon for deep emotional state. Happiness is a choice because thinking and focusing positively and learning from negative experiences can provide a success-oriented increase in happiness level.

Today, it is said that feeling energized, a sense of belonging and a sense of purpose are the most important things for employees, and getting a fair wage is the least factor that really makes a difference. Possibly, this also means that it will be more difficult to be happy if one is very underpaid or living in poverty or living in working poverty, as he feels that he is not actually respected and honored through his salary (Fisher and King, 2022). As a matter of fact, salary really has little importance in the business environment. An employee with a very high salary may not be happy in return. The factors underlying their effect on their performance and happiness can be exemplified as the type of work they do, the personality traits of themselves and their manager, and characteristic harmony with the requirements of the job (Misra and Srivastava, 2023). In addition, according to Lyubomirsky (2011), 50% of happiness depends on genetic factors, 40% on purposeful activities and 10% on living conditions.

Among the indicators of happiness at work, enjoying the work environment such as associating successful completion of especially challenging projects with personal satisfaction, a sense of completion, feeling peaceful, and feeling cared for, conceptualizes the pursuit of happiness with both hedonic and eudemonic aspects. Employee happiness is the satisfaction that employees experience at work (Kaul, 2014; Brogan, 2023).

Although they cannot measure employee happiness exactly, for accurate estimation, methods such as organizing regular surveys to get feedback on issues such as job satisfaction and loyalty of employees, tracking workforce turnover and retention rates to measure their loyalty, measuring their productivity and performance to evaluate their commitment and motivation, encouraging open and honest communication with them to provide feedback and get their improvement suggestions can be used. Organizations should care about measuring the dose of employee happiness because investing in employee well-being can in turn support productivity and positive progress by all parties (Brogan, 2023).

While even chocolate does not create happiness anymore due to reasons such as child labor and dangerous working conditions (Kalfaoğlu, 2022), it is necessary to evaluate happiness in terms of morality. Happiness is the template for a moral life. Happiness suits good people, as Aristotle said. Eudemonic happiness also appears exactly in the way that the individual acts under the guidance of the mind with his moral values and ultimately develops his own virtues (Cederström, 2015). In this context, the importance of mental inquiries in the moral-happiness-virtue spiral is undeniable. However, the level of happiness is another important factor to consider.

## 3 The harmful aspects of overdose happiness

Happiness is seen to be usually associated with feelings such as pleasure, satisfaction and pleasure. Generally, focusing on the presence of positive emotions has led to ignoring negative emotions while conceptualizing happiness (Joshanloo, 2014). However, positive emotions such as joy, happiness and love are not always pleasurable, but can also be considered as feared emotions (Gilbert et al., 2014). However, it is thought that cultural values such as the evil eye, the beliefs that exist in the society regarding this issue and some religious values may also be the underlying reasons for fear of happiness. For example, in Asian culture, happiness is seen as an unpleasant or even feared emotion at some times and in some contexts (Joshanloo et al., 2014).

On the other hand, the pursuit of happiness is also tiring. Living with a focus on happiness actually imposes a responsibility that can never be fully fulfilled. When one makes happiness a duty, the failure of the person to succeed in the end may cause the individual to feel worse. Therefore, this search can actually make the person much unhappy and lonely (Spicer and Cederström, 2015).

We have also witnessed an increase in motivation seminars in organizations in recent years. There are managers who see motivation as companions in line with happiness and productivity. Some organizations (e.g., Google) are even supposed to have a happiness coach (or chief). They help employees become better than they are, such as being healthier, more productive, more cheerful, and kinder. In other words, a parallel increase in happiness and other positive job and employee outputs is expected. In fact, the goal of increasing the happiness and productivity of employees has been one of the issues that managers have been obsessed with for over a century. The Hawthorne experiments conducted by Elton Mayo and his team from the mid-1920s to the early 1930s pursued this goal. On the other hand, the information that was thought to be clear in the current century seems to be blurred or the factors that form its basis and result seem to have shifted. Evidence has been found that these steps in the pursuit of happiness do not always and everywhere offer a good idea. Some studies have predictions and conclusions that happy employees will become unproductive after a while, as opposed to creating myths. For example, a study conducted on employees in British supermarkets revealed that there are negative relationships between satisfaction and productivity, and that the much unhappy their employees are, the better corporate profits (Spicer and Cederström, 2015). Wittman (2017) also states that managers should stop trying to make employees much happy, develop their “personal resilience” in obtaining the commitment they want, so that they will have more persuasion power for every difficult task.

Employee commitment and belonging in organizations is one of the most desired issues for managers. The productivity of the engaged employee is undoubtedly higher (Sgroi, 2015). However, although they have relations with each other, employee engagement, which is defined as “employee’s dedication to work and using himself/herself physically, cognitively and emotionally at work” (Kahn, 1990), should not be confused with employee happiness. The happiness of the employee may not result in working hard for the organization. He can achieve happiness by using the resources of the organization, but this situation may not be reflected in his performance, especially in public organizations (with the belief that the state’s resources are unlimited) and the situations encountered because of unqualified job recruitment, for example. Focusing on and chasing happiness can have various side effects (Lee, 2020). In fact, Mauss et al. (2011) expressed a paradox that people will be disappointed to the extent that they value happiness. According to them, happiness should not be a goal such as succeeding in an exam or getting a desired job, because it is an abstract feeling and cannot be measured.

Moreover, it is utopian to expect the employee to be happy all the time. This expectation may cause the employee to act fake and actually be less happy. Actually, a study conducted in Germany found that pretending to be happy at work increases stress and can cause various health problems from depression to cardiovascular disorders (Duke, 2006). The fact that the employee does not behave as they are and is forced to constantly control their emotions, experiencing stress, and indicators such as poor management and toxic culture may herald that things are not going well in terms of management and functioning (Lee, 2020), it also shows that the person has a high level of self-control and resilience and can also help a person get promoted to higher positions in a competitive business environment. In fact, research has started to give more importance to the importance of masking emotions in the workplace. They attributed the success in business life to the fact that negative emotions should be told less intensely than they are actually felt, or not at all, and they stated that hiding one’s emotions actually has the ability to manage their emotions effectively (Sarro and Curl, 2021).

## 4 Manager-employee relationships with personality traits and dependency perspectives

An overdose of happiness in business life does not always work. For some jobs that require specific skills, a person’s characteristics affect their ability to do the job. The inverse relationship between performance and happiness becomes even more evident in such jobs. One study (Van Kleef et al., 2004) found that angry people perform better when negotiating than happy ones.

At the center of happiness is awareness. The mastery of the manager lies in the perfection of the overlap between self-awareness and situational awareness (Wagner, 2016). A picture of mutual excellence emerges when a manager who applies the right management and organizational techniques to achieve a certain goal makes the right job match with the talents of the employees. The manager is happy, the employee is happy. The improvement in the employee’s performance makes the manager happy and satisfied (Matagi et al., 2020, 2022).

For some managers, being happy all the time is not very important or necessary. Scenarios can be created for reasons that will make employees happy: for example, to work less, to earn more money, to go to work less. Managers focus more on employee performance and productivity. In this case, employees who are the cogs of the machine feel obliged to keep their performance high under intense control. They like to be worked hard and prefer to use negative feedback to encourage it. The result of a study showed that about 1 in 8 workers (13%) are clearly discouraged by their employers from taking a break, and more than a quarter (28%) regularly work until lunch. In addition, although 1 in 3 (31%) employees spend more than 4 h a day in meetings, one in five (19%) stated that they are uncomfortable taking breaks because their managers may find them unproductive (Dunaway-Seale, 2022).

Leadership is contagious. Their emotions, mindsets, and behaviors ripple around them. In this context, every emotion felt has a common responsibility. In the arena of challenge, the ambitions of the leaders pass on to their employees.

With their ambitious and competitive nature, workaholics need to work excessively even if it risks their health, happiness or socialization (Oates, 1971; Snir and Harpaz, 2009). They expect to be engaged, immersed, and self-actualized at work. If they are not satisfied and happy with their job or career, they interpret this as an indication that they need to change (Chamorro-Premuzic, 2020). This is exactly what ambitious managers want! As a matter of fact, Illouz (2007) revealed that the effect of work-related happiness is detrimental in his negotiations with people who spend their private lives using the methods they have experienced in their organizational life, who make their home life colder and more calculating, and who want to spend their lives at work rather than home.

For most employees, work cannot be personalized and is essential to make a living. Money earned by working indicates a comfortable life. In addition, working is a reflection of being a responsible member of society. In the competitive business environment, the main concerns are profit, supply, demand, efficiency and productivity. Although the factors that make employees happy are largely due to their personality traits, managers may have to choose between a happy or productive employee in certain periods, especially during economic crisis. The concern that a state of psychological well-being that will create intoxication such as happiness will be permanent disturbs the manager. From another point of view, with the perfection of matching the employee to the right job, the employee tries to reveal his full potential and serves the purpose with all his productivity. His concentration works intensely. The constant preoccupation of his mind leads to loss of self-consciousness and deterioration of the sense of time he feels (MacMillan, 2009; Misra and Srivastava, 2023). This type of personality, called the autotelic personality, seems to be attributed to the employee who has exactly the qualities sought for an ambitious leader.

On the other hand, being dependent on the manager together with work dependency can harm both relationships and the person himself. Employees who have to face emotional neediness always need to be recognized by their managers, making them overly emotional and reactive (Ekman, 2013). Additionally, as in the “Pygmalion effect” (Livingston, 1988), employees who feel that their managers have high expectations tend to show higher performance even when faced with challenging tasks. For those who see their managers as a source of inspiration, losing their job can lead to the most devastating situation of their lives. Because, whatever the reason, their dismissal causes them to lose not only their sources of income but also their sources of happiness (Sennett, 1998). In the fight against emotional addiction, unrequited happiness seems out of the question. Happiness takes a long way to build mutual responsibility and trust.

In the psychology literature, excessive dependence on people is referred to as Dependent Personality Disorder (DPD). Because of addiction, people’s ability to make decisions or do things on their own weakens. This stickiness to others around them can also lead to unhealthy relationships. They also lead a very depressed and anxious life. It is actually possible to turn the manager-employee relationship dependency in favor of productivity. Environmental support is very important here. Making the person feel valued, being supportive rather than directing their decisions, giving positive feedback on their actions alone, making them feel safe and not being judgmental will have a positive effect on the treatment of relationship addiction.

## 5 Benign stress and its effect on happiness-performance relationship

The issue of stress in business life from almost all aspects (e.g., social, organizational, individual, environmental, etc.) has been addressed in the management literature and continues to be important (Eddy et al., 2023; Nimmi and Donald, 2023; Raza et al., 2023). It has been reported that job stress is an individual’s harmful physical and emotional responses due to the mismatch between tasks and environmental requirements and the employee’s needs, resources and abilities. However, it is necessary to separate the stress as malignant and benign in itself in explaining employee performance. In addition, it is necessary to emphasize the aspect of benign stress that leads the person to achieve the goal, when he enjoys working, during the completion of a project on which the stress is focused, or while completing any task that falls on him. Yerkes and Dodson’s (1908) Inverted-U Model has been an important study in terms of enabling organizations, managers and leaders to see the sometimes beneficial and sometimes harmful aspects of stress. Beneficial stress is harmonious and constructive, whereas destructive stress contains maladaptive and harmful features (Örücü et al., 2011). While the motivating effect of constructive stress increases the commitment of the employees to their work and makes them do their jobs voluntarily, firstly the individual and then the organizational performance, destructive stress brings with it results that the organizations do not want as it prevents the employees from doing their duties (Eren Gümüştekin and Öztemiz, 2005). Managers and leaders will gain the opportunity to increase performance when they notice the workloads of the employees and the pressure they experience while they distribute the tasks and realize whether new tasks will put them under pressure or whether the pressure will be sufficient (Turgut and Turunç, 2017).

The work of Loureiro et al. (2023) is also valuable in this regard. They argued that the interaction of artificial intelligence technology and benign stress could increase happiness in the workplace. Similarly, Mauss et al. (2011) found that while people value happiness, they are less happy when exposed to low-stress conditions. Therefore, it is possible that situations such as benign stress that add dynamism can be associated with more happiness.

## 6 The inverted-U model of employee happiness

An overdose of anything is harmful. Overeating, excessive leisure time or excessive workload… Or rather, an overdose of everything is unhealthy, just like happiness. In fact, there are studies that present numerous examples on this subject. For example, Pathak (2022) suggested that excessive exercise damages the joints, sleeping more than the recommended 8 h causes heart problems, and drinking too much water can cause death. Riggio (2013) also attributes the bad turn of the good to personality traits, skills and abilities. Someone with too much self-confidence may be arrogant and narcissistic, someone who is extremely conscientious, a perfectionist, and someone who is overly intelligent may lack communication (Sugay, 2019). Essentially, it is important to experience important positive moods, such as happiness, in moderation. As Gruber et al. (2011) stated, happiness has its price and negative emotions have a valuable place in life, too. For them, three positive emotions (such as joy, gratitude, or hope) create a good balance for each negative emotion (disgust, shame, fear).

In the literature, there are studies that address the harmful aspects of overdose of happiness in business life. For example, the study of Lam and Gurland (2008) found that happiness makes people productive at work, but only up to a point. After a certain threshold, being too happy creates a lack of motivation, which is never desirable for managers. Researchers have investigated how happy they are with hundreds of employees, as well as how often they perceive themselves to be engaging in proactive behaviors such as talking about and problem solving. It has been explained that when everything is thought to be in order, employees cannot be motivated to make improvements. Indeed, the importance of motivation in business life is of great importance for the continuity of activities, even if it is an already highly successful organization. Its absence or scarcity is worrisome for the future of the organization.

Psychologists Yerkes and Dodson (1908) in their studies on the effects of stress on the decision-making abilities of mice, found that the responses vary according to the stress intensities, and they used the Inverted-U Model (The Inverted-U Model, also called Yerkes and Dodson’s Law) (Turgut and Turunç, 2017). Using this model, for example, Anderson (1990) and Levitt and Gutin (1971) have shown that too high or too low arousal or stress level will significantly reduce performance. According to the model, the most appropriate stress will provide the highest performance (Örücü et al., 2011). As Schermerborn et al. (1988) stated, it is seen that employees are careless and unwilling toward their work due to low performance under low pressure, as on the left side of the model, and low performance under high pressure, as on the right side. On the other hand, in the middle part of the graph, it is seen that efficiency and effectiveness increase with dosed stress and balanced arousal under balanced pressure. The model suggests that the relationship between stress and performance will initially show a positive relationship type at a certain arousal or a certain stress level, and a negative relationship type after a certain level is exceeded (Eren Gümüştekin and Öztemiz, 2005).

**Figure 1:**
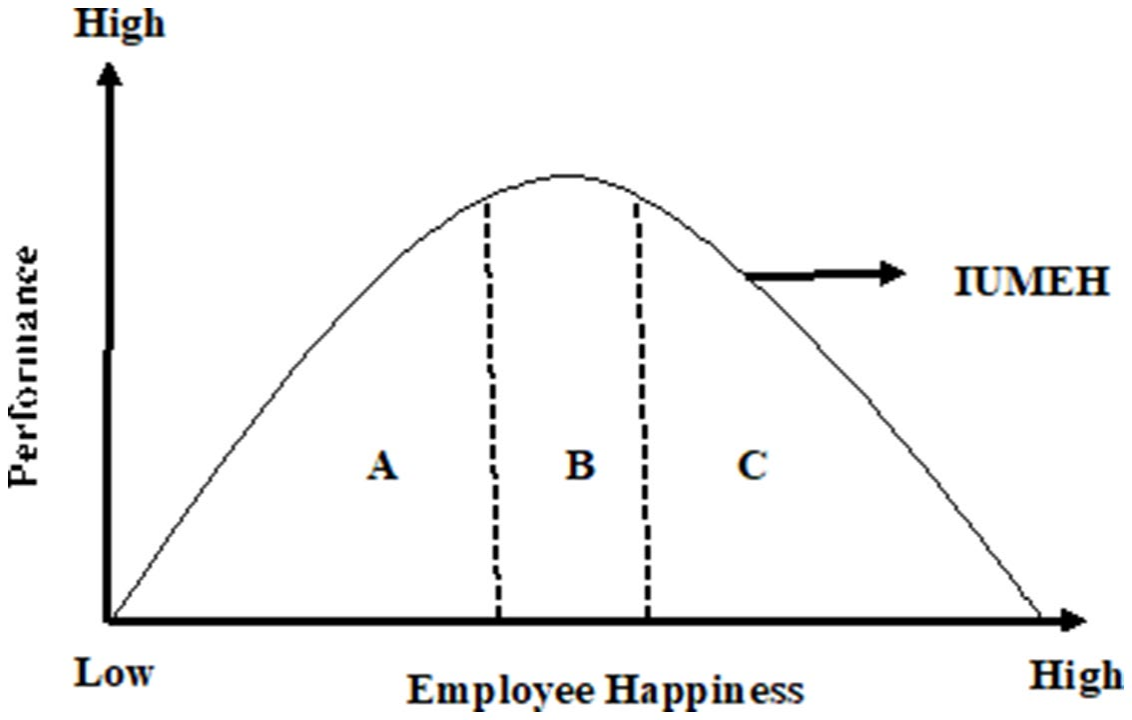
The Inverted-U Model of Employee Happiness (IUMEH). **(A)** Supporting Happiness. **(B)** Reflecting Happiness in a Balanced Dose. **(C)** Negative Effect of an Overdose of Happiness (or Happiness Intoxication) on Performance. Adapted from Yerkes and Dodson (1908).

Inspired by Yerkes and Dodson (1908), the “Inverted-U Model of Employee Happiness (IUMEH)” was developed in this study (Figure 1). Shown as A in the model is the area that supports happiness. As the individual outputs of this area can be exemplified as follows: the increase in the welfare level of the employee (earning above the average), improvement in working conditions (e.g., the harmony of working hours with the person, the balance of authority and responsibility, sensitivity to opinions, suggestions, requests and complaints, etc.), balance with family life, increase in social life quality (increase in social networks, balanced time spent for fun, effective time management), harmony between personality traits and the type of work done and its requirements, increase in motivation level, increase in satisfaction level, unity of purpose, excitement. On the other hand, organizational outputs for an area can be exemplified as an increase in efficiency, effectiveness and productivity levels, organizational peace, and increase in organizational competitiveness, and decrease in employee turnover rate. The area shown as B in the model describes the area that reflects a balanced dose of happiness. With the presence of sufficient arousal, it is seen that happiness reaches a balance in this area and performance is at its peak. In the C area, where happiness reaches an overdose, the relationship between happiness and performance evolves into a negative relationship. Intoxicated with happiness, the person encounters factors such as lack of attention and care, reluctance to take risks, reluctance or delay in completing the current task, unwillingness to start a new task, lack of motivation, shyness in taking responsibility, tendency to be deceived quickly, stress, desire to change jobs. Possible organizational consequences after overdose of happiness can be exemplified as decrease in performance, decrease in productivity, efficiency and effectiveness, increase in employee turnover, and decrease in competitiveness. The Employee Happiness Curve (IUMEH) becomes frightening for managers as happiness decreases other positive variables (Area A) after a certain point (Area C; Figure 1).

A systematic literature review was used to exemplify individual and organizational outcomes in each region of IUMEH, following the adopted methodological approach. This methodology seems to provide exclusive evidence for IUMEH’s assumptions. The use of this approach is valuable as it reduces author bias and enables a more detailed examination of the research question (Çınar, 2021). This study followed the methodology process in the following order and for the following reasons:

Firstly, to what extent and in what ways are employee happiness and job performance related? The research question is clear, concise, and well-structured. To ensure a high-quality systematic review, it is important to prioritize ‘why’ before ‘how’ of research. This approach is essential for evidence-based practices. Additionally, the PICO(S) model was used to narrow down the subject and find relevant evidence for the research questions (Khan et al., 2003; Schardt et al., 2007; Davies, 2011; Uman, 2011; Yannascoli et al., 2013). Information about employees is included as *Population*, individual and organizational factors affecting the happiness of employees as *Intervention*, scientific publications comparing the happiness levels of employees in business life and individual job performance levels as *Comparators*, proven results of relevant publications as *Outcomes*, and information about the study designs used as *Study Designs*.

Secondly, studies that deal with the components of the determined research question, first “employee happiness” (One Hundred and Two, URL-1, 2024) and then “employee happiness” and “performance” together (Sixty, URL-2, 2024), without any limitation on the year of publication were examined, but when studies that did not indirectly examine the relationship between employee happiness and job performance were excluded, a protocol was wanted to be created with 35 studies. Since there were concepts that could be considered synonymous during the research, for example, using the concepts of “worker happiness” instead of employee happiness or “individual performance” instead of performance was a guide in expanding the scope of the research. Because there were only seven studies that examined the variables “employee happiness” and “job performance,” which constitute the main variables of this study, together (URL-3, 2024).

**Figure 2:**
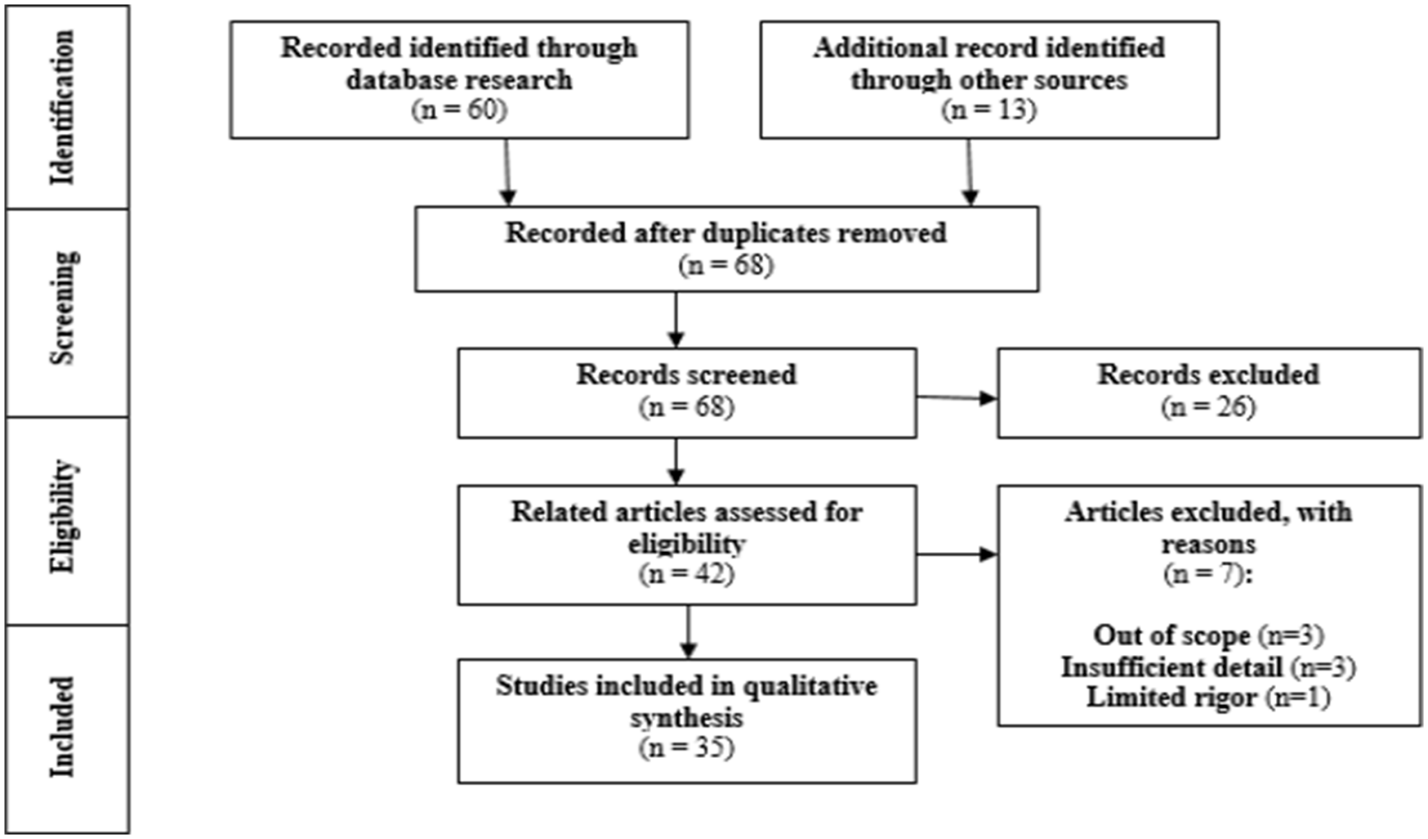
PRISMA Flow Diagram (Moher et al., 2009) of the study.

Thirdly, in order to use a clear and reproducible method, the keywords determined in the previous step were preferred to search the pre-finalized WOS (Web of Science) database. The main reason for choosing the relevant database is that it allows accessing the most relevant and prestigious publications in the field of research by regularly scanning the journals in the relevant field of science. In addition, it was decided to include gray literature (Rothstein and Hopewell, 2009; Adams et al., 2016) and reference lists with a snowball approach in the scanning, using the PRISMA (The Preferred Reporting Items for Systematic Reviews and Meta-analysis) (Moher et al., 2009) standard during the scanning (Yıldız, 2022). Selecting more than one screening area in order to find more evidence of the operating model for those concerned complies with the standard in question. The PRISMA Flow Diagram of the study is shown in Figure 2.

Fourthly, the synthesized findings of the studies, especially in WOS, are presented in tabular form, in accordance with PRISMA rules, to evaluate the validity and quality of the appropriate data (Table 1). Gray literature and snowball approach references are generally summarized in the study, and their compatibility with the relevant regions in IUMEH is included in the last column of the table.

**Table 1:** Systematic literature review on employee happiness-job performance relationship.

Area represented at IUMEH	Related database	Authors and related journal	Topic [Other than demographic variables and employee happiness (EH)]	Sample	Related finding(s)	Gray literature and some other references
A	WOS	Bingqi et al. (2023)^+^ (Social Behavior and Personality: An International Journal) Choudhary and Kunte (2023)* (Employee Responsibilities and Rights Journal) Hafeez et al. (2023) (Journal of Management Development) Kaur and Kaur (2023)^+^ (International Journal of Indian Culture and Business Management) Alahbabi et al. (2023) (Quality-Access to Success) Bellet et al. (2023) (Management Science) Bani-Melhem et al. (2020) (International Journal of Productivity and Performance Management) Thompson and Bruk-Lee (2021) (Applied Research in Quality of Life) Chang and Hsu (2015) (Qual Quant) Dasgupta et al. (2014) (Corporate Communications: An International Journal) Howard and Gould (2000)^+^* (Am J Ment Retard)	Corporate Social Responsibility (CSR), Financial Performance, Altruistic Values High-Performance Work Systems (HPWS), Work Intensification (WI), Organizational Growth (OG) Job Variety, Stress, Employee Engagement, Burnout Factors affecting EH Servant Leadership (SL), Job Performance (JP) Productivity Leader-Member Exchange (LMX), Employees’ Innovative Performance, Service Climate Job Demands, Organizational Commitment, Job Performance, Turnover Intentions Employee Information Literacy (EIL), Employee Creativity Performance (ECP) Managerial Communication Practices, Job Performance, Commitment, Absenteeism, Turnover Intention Organizational Performance, Strategic Planning	286 healthcare professionals 70 paper from 2000 to 2023 in Google Scholar, Emerald Insight, Scopus 169 nurses in Pakistan A hybrid approach = Approximately 100 research articles + focused group interviews (FGD’s) and semi-structured interviews Total employees and administrative staff working in Abu Dhabi Health Services Company (SEHA) are 18,759. But no information about the sample. 1,438 sales workers from BT in the United Kingdom 303 frontline employees in service organizations in UAE 222 participants who are over the age of 18, work in the USA, and spend at least 90% of their working hours in an office space 355 employee in Industrial Technology Research Institute in Taiwan 101 employees in three manufacturing organizations in eastern India Discussion	CSR increases financial performance in healthcare organizations, EH has a mediator and altruistic values have a regulatory role in this relationship. An integrated model including HPWS, WI, EH and OG has been developed. Job diversity has increased both employee engagement and happiness, as well as burnout and stress. With its two-way impact, job diversity should not always be seen as a positive job resource. Factors such as work environment, reward and recognition, leadership, and work-life balance are the ones that affect EH the most. EH is the mediator in the relationship between SL and JP. EH was examined by taking advantage of the variability in weather exposure along with workers’ mood, and it was ultimately proven to have an impact on sales performance. Since high levels of LMX will increase individual attention and care in employees, EH also increases. At the same time, it is not clear whether the high service climate can be linked to attention to the needs of them. Increasing job demands have reduced employees’ happiness, commitment to the organization and performance, while increasing their intention to leave work and inefficient work behavior. As the level of perceived happiness increases, employees’ creative behavior increases, and EH regulates the relationship between EIL and ECP. Some aspects of managerial communication such as collaborative approach, respect/recognition, flexible working arrangements, trust, clear direction, involvement, and autonomous and challenging tasks are beneficial for both EH and JP. Employee happiness should not be considered as a business goal, a strategic plan should be created accordingly.	Brogan (2023) and Fredrickson (2004)
B	WOS	Potipiroon (2024)^+^ (Journal of General Management) Erselcan and Süral Özer (2023) (International Journal of Contemporary Economics and Administrative Sciences) Arshad et al. (2023) (Frontiers in Psychology) Wright (2014)* (Journal of Organizational Behavior) Walczak (2014) (Knowledge for Market Use: Media and Communication in the 21st Century)	Job Satisfaction (JS), Job Performance (JP), Interpersonal Justice Climate (IJC) Job Satisfaction, Cultural Characteristics Organizational Virtuousness (OV), Emotional Intelligence (EI) Objective Indicators, Eudaimonic Well-Being, Life Satisfaction, and Emotion-Based Well-Being Eudamonism, Basic Psychological Needs (Autonomy, Competence, Relatedness), Core Self Evaluations, Person-Organization Fit, Job Satisfaction	192 employees in a public organization in Thailand 409 Italian and 550 Turkish employees 416 bankers in Pakistan An overview of the literature on happiness 1,021 working adults in Poland	Although job satisfaction is thought to always create high job performance as an indicator of employees’ happiness at work, the effect of JS on JP is significant when IJC is low. According to the result in the Turkish employee sample, EH and JP are positively related, but the relationship between job satisfaction and performance moderates this result. Regarding EH in terms of subjective well-being, there is a mediating effect between EI and OV. Emotion-based well-being is more dominant than the other three facets of happiness in interpreting relationships such as JP, and especially in increasing satisfaction-based correlations. As Herberg suggests in his dual factor theory, the work environment should be reorganized to offer the individual more autonomy and feedback and enable employees to discover their talents. Employees who know the results and importance of work are happy and indirectly try to improve their performance. In terms of the edudaemonist philosophy of life, EH is determined by harmony perceived by employees and their basic psychological needs.	Dunaway-Seale (2022) Davis (2009), and Lam and Gurland (2008)
C	WOS	Met et al. (2023) (Heliyon) Gutiérrez et al. (2020) ^+^** (Span J Psychol.) McCarthy et al. (2016) (Journal of Applied Psychology) Hsiao et al. (2015) (International Journal of Contemporary Hospitality Management Rego and Cunha (2009) (Journal of Happiness Studies) Wright and Cropanzano (2007)* (Research in Personnel and Human Resources Management	Performance (or Success) - Workplace Anxiety, Emotional Exhaustion, Cognitive Interference, Supervisor-rated LMX, Peer-rated Coworker Exchange (CWX), Supervisor-rated Job Performance (JP) Supervisor support, Quality of interpersonal relationships, Physical work environment, Peer conflict, Demands of teamwork, In-role Performance (IRP), Customer-directed extra-role performance (CDERP) Individualism–Collectivism Orientations, Affective Well-Being (AWB) Job Retention, Job Satisfaction, Psychological Well-Being,	4,277 bank employees 43 articles in some databases such as WOS, SCOPUS etc. 595 police officers in Canada 247 Hospitality Frontline Service Employees and 43 their managers in Taiwan 161 employees of 109 organizations in Portugal Discussion	Although the curvilinear relationship between happiness and performance is inevitable, the pace of happiness decreases once a certain level of success is exceeded. From a well-being perspective, there are four types of EH and six types of JP, and the relationships between these types lead to different results. Additionally, as EH increases, there is no single type of relationship that will necessarily increase JP. The relationship between employee mood and JP is more sensitive to LMX than CWX. When LMX is strong, employees hide their negative moods and their performance is staggering. There is no a strong symmetric relationship between EH and performance assessments. Also, “high EH alone is insufficient in explaining high quality of work as assessed by managers.” It is assumed that when employees’ happiness increases, their performance also increases. However, based on cultural differences, high collectivist tendencies may not always parallel high AWB feelings. The Happy/Productive Employee thesis has been evaluated with the satisfaction and psychological well-being included; job satisfaction is an incomplete interpretation of the relationship since it only describes job-related aspects. There may be differences in performance between the psychological state of the employee and the job.	Lee (2020) Spicer and Cederström (2015) Snir and Harpaz (2009) Lam and Gurland (2008) Illouz (2007) Duke (2006) Van Kleef et al. (2004), and Oates (1971)

1. The studies in each region are listed by year, from the most current to the oldest. 2. +Since it is not open access, only its abstract text has been examined. 3. *Review Article. 4. **Meta-Analysis and Scientific Mapping.

Finally, a critical discussion is presented along with the results and interpretations, and the inferences are supported. Based on the transparent process and justification of all selections, the study can be considered reliable and reproducible, depending on the inclusion criteria. It is important to consider these factors when evaluating the methodology’s quality (Pati and Lorusso, 2017; Figure 2).

As seen in Table 1, examples are presented from researchers who offer homogeneous, heterogeneous, parallel or inconsistent views on each field at IUMEH. Considering different variables in interpreting the relationship between EH and JP helped to delve deeper into the subject. In addition, such awareness is a guide for future researchers. With the increase in applications such as CSR, HPWS, managerial communication in organizations, long-term interventions that require processes such as LMX and WI, and approaches that increase the dynamism of work environments such as job variety and job demands, EH seems to have a mediator effect on JP. However, it is understood that EH, along with concepts such as IJC, job satisfaction, OV, EI, have an indirect and moderating effect on JP. According to some researchers who argue that the performance of every happy employee cannot be high, the change in the levels of the concepts included in the model is important. Moreover, the sensitivity between variables may vary depending on the field, sample and sector of research. It has been stated that EH and performance evaluations cannot be subject to symmetrical relationships.

In general, studies in the literature on happiness emphasize that each level of happiness yields valuable results for both employees and organizations (Scott, 2008; Carr, 2011; Singh and Aggarwal, 2018). Furthermore, John Stuart Mill’s Utilitarian theory (1861) states that it is essential to calculate the impact of happiness on different courses of action and determine the one that results in the greatest happiness. Utilitarians must also measure happiness and identify those who are affected by a specific activity (Buckley et al., 2001). Similarly, Cartwright and Holmes (2006) suggest that happiness can be experienced at three levels: ‘a pleasant life’, ‘a good life’, and ‘a meaningful life’. They argue that while a pleasant life may provide temporary happiness, a meaningful life provides longer-lasting happiness. Gullekson and Dumaisnil (2016) analyzed the emotional states of individuals based on their status level. They concluded that individuals with lower status are more likely to experience negative emotions such as anger, boredom, sadness and fear, and less likely to experience happiness compared to those with higher status. Additionally, the study found that HRM systems can improve happiness and relationships but may have a negative impact on health. Therefore, calculating the measure and effects of happiness is an important issue as well as the emergence of the components associated with happiness (Kırpık, 2020).

Although happiness cannot be precisely measured and even its definition as a momentary change of mood is still not satisfactory, implications are presented with this model on possible indicators that help to understand overdose. It should also be noted that the curvilinear aspect of the model may differ in terms of culture, type and characteristics of the business, private, public or non-profit enterprises, the generations of managers and the level of managers (such as upper, middle, lower).

Cultures emphasize different orientations (for example, individualism and collectivism) that lead to significant changes in people’s feelings, thoughts, and behavior. For example, psychologists have shown that fear of happiness – the belief that happiness has negative consequences and should be avoided – is stronger in collectivist/collective cultures (Joshanloo and Weijers, 2014). Culture plays a role in societies being collective or individual, and this individuality or collective being affects the feelings, thoughts and beliefs of individuals (Çevik, 2020). Fear of high happiness causes collective cultures to be less concerned with positive emotions (Bryant and Veroff, 2007). They believe that positive emotions will bring negative results, and such individuals are afraid of positive emotions and stay away (Joshanloo, 2013; Muhtar, 2016; Lambert et al., 2018). Collective cultures, where the fear of happiness is high, teach individuals to be afraid of happiness with proverbs, idioms, songs, stories and similar ways (Joshanloo and Weijers, 2014; Özdil, 2017). It is stated that there are opinions that the fear of happiness may be more common especially in eastern cultures (Bryant and Veroff, 2007; Joshanloo, 2013).

Joshanloo and Weijers (2014) tried to explain the voluntary inhibition of happiness based on belief cultures in their research and examined this system in three dimensions: (1) Happiness is not a permanent state of emotion, (2) Belief values tend to prevent or prevent the happiness of individuals who make up the society, (3) It has been understood that the fear of happiness is a necessary condition. According to researchers, some individuals think that happiness is a characteristic of bad people, expressing happiness is a bad behavior, and seeking happiness is bad. In addition, the authors state that people from different cultures tend to avoid feeling happy and being happy for different reasons, and the degree of this varies from person to person. One of the reasons for this is the belief that excessive happiness will have bad consequences. It is the thought that there is a high probability of encountering undesirable negative things immediately after being very cheerful or achieving happiness. It is stated that there may be a state of dislike for happiness because happiness is seen as a symptom of bad things for both oneself and those around him (Türk et al., 2017).

In terms of job types, this curve has a high impact on employment contracts with an indefinite duration. There will be no need for a long-term interpretation of employee performance, since there will be no continuity of work in employment contracts concluded depending on objective conditions such as the existence of a certain period and the completion of a certain work or the emergence of a certain phenomenon.

In terms of private, public or not-for-profit enterprises, it seems that private enterprises and their managers, which benefit most from performance evaluations and which can be described as the most brutal among them, have a larger share in the competitive market than others, and their managers are more likely to benefit from IUMEH. They ensure that the sanction clauses related to employee performance are included in the contracts in the axis of labor law, and a kind of invisible hand has performance pressure on employees.

The effect of the management style chosen and implemented by the manager on employee performance has been proven for years. Although the perspective of the employee as the cog of the machine has changed since the Taylorism period, the burden still rests on the shoulders of the employees in terms of organizational efficiency. However, depending on the personality traits, the manager’s relationship with the employee and the level of communication bonds change. For example, managers belonging to the X generation, who are known to have characteristics such as facing economic recession periods, meeting with technology later, being purposeful, endless respect and trust in authority, and loyalty to rules, have a high level of motivation and performance expectation from their employees in accordance with their management style. This generation, which has struggled with poverty, also expects employees to be contented and cautious. The next generations were introduced to popular culture and media, and grew up in a living space with a higher level of well-being and self-confidence. Therefore, employee expectations will differ from previous generations.

Moreover, acknowledging cultural, gender, or industry-specific differences in the experience and management of employee happiness would broaden the scope of IUMEH. In cultures where happiness is believed to be bad (e.g., Eastern cultures such as Iranian, Japanese, Chinese, Turkish), the state of happiness is attributed to not having faced the painful points of life and not having encountered its tragic consequences, and it is argued that excessive happiness will result in not being able to reach personal maturity. IUMEH may be observed more frequently in organizations in such cultures. Because in these societies that want to become a part of a whole, individual and momentary interests should not prevail over organizational interests. On the other hand, the societies of the Scandinavian countries (for example, countries such as Iceland, Finland, Sweden, Norway and New Zealand) are helping more employees to be happy and especially to women’s career mobility, thanks to effective social policies on issues such as gender equality, social support and family-work balance. Gender-based employee happiness also varies across cultures. For example, in research conducted by the state statistics agency in Turkey between 2017 and 2021, women are happier than men in every period. The pressure put on men by the crises experienced in those years is stronger because in this society, the person who is primarily responsible for family income is the man (Gök and İl, 2017; Akgül, 2022). Therefore, gender-based differences may also create differences in the impact of IUMEH. Moreover, the working environment and the sector that adds value also share in this impact. In the study conducted by BambooHR by analyzing the data of more than 1,600 businesses and nearly 60,000 employees around the world between 2020 and 2023, the construction, technology, finance, non-profit organizations, food, travel and accommodation, education and health sectors were the most important sectors, respectively. These were sectors with high levels of happiness (Üren, 2023). The fact that the average hourly wages of employees were higher than in other sectors and the differences in personal and sectoral priorities along with the Covid-19 pandemic were effective in this result.

Finally, it should be stated that the model might differ according to the functional and hierarchical level of the managers. It is predicted that middle-level managers who give priority to corporate goals will benefit more from the model compared to the top managers who determine the organizational strategy and lower level managers who manage the daily tasks of the employees. Middle-level managers are the internal intermediary of the organization in financial and social audits, as they are concerned with plan development and decision-making in their area of responsibility and are responsible for the implementation and supervision of the plans.

It is useful to mention the following two theories that help explain the model. The first of these is the Behavioral Change Model of Kuhl and Atkinson (1986) and the second is the Flow Theory developed by Csikszentmihalyi (1990).

The Behavioral Change Model defines the word motivation as “change in behavior.” According to this definition, there is a relationship between intent and action. Researchers using the behavioral change model mostly focus on the concept of “will” (volition). The results obtained because of the examination of this concept, motivation and will constitute the driving force that the person sets his own goals and uses to pursue his own goals. For example, if a person is motivated to be self-sufficient, the will to be self-sufficient (volition) arises as a side element of this motivation. This couple, that is, motivation and will, can be thought of as intention and it can be accepted that intentions turn into actions in different ways at every step (Şeker, 2007). Kuhl and Atkinson (1986) have studied this transformation in three ways, and changes in behavior can be modeled using these three basic elements:

1. *Instigation:* If an action has the feature of satisfying the motivation of the person, the tendency toward this action increases.
2. *Inhibition:* If there is a factor/obstacle preventing an action, the person’s tendency to action decreases.
3. *Consummation:* This motivation loses its power when a person performs any action in the situation.

It is also important to evaluate the model with the flow theory developed by Csikszentmihalyi (1990). First, although a lot of work is done on a job, it is seen that the same motivation and productivity are not effective in completing the job. Probably that job does not describe the same intensity of emotion for the person. The important point here is that even if the individual does not change or the nature of the job remains the same, these differences can occur. People can interpret their work differently. Flow experience occurs when the person is fully focused on a job, the difficulty level of the job and the person’s ability in that job are high. Flow Theory is that a person is immersed in that job while doing a job participates in the process, does not notice how time flies, and lives that moment intensely. The high interaction and harmony of the characteristics of the work with the person’s work helps to complete the work without understanding how time passes and to enjoy the present moment (Özşahin, 2005; Kiremitçi Canıöz, 2021).

There are also two theories used in the development of IUMEH. According to *Affective Events Theory* developed by Weiss and Cropanzano (1996), the relationships between individuals in business life and their jobs affect their daily and professional lives. Employees’ emotions and moods are at the center of their professional lives (Donald et al., 2016; Başar, 2022). If the characteristics of the organization and the qualities of the job allow certain events to occur in the work environment, the happy and sad events that employees experience in that work environment lead to positive or negative reactions with the influence of the employees’ personalities and emotional states (Weiss and Cropanzano, 1996; Ateş, 2017). According to theorists, the ability of employees to perform the tasks they are asked to do in the best way, especially in labor-intensive jobs, is related to the emotions they feel. From this perspective, it is stated that employees in lower-level positions show stronger resistance to negative events (Basch and Fisher, 2000). In addition, the existence of employees who experience the same event and give different emotional reactions shows that they change only according to their current emotional state and personality traits. With this theory, it is understood that IUMEH is affected by the emotional events experienced by employees. It has been observed that it supports the area A, especially with the assumption that positive events will lead to positive reactions, and it supports the area C, which will be adversely affected by the individual’s current emotional state and personality traits components.

On the other hand, *Emotion Regulation Mechanism* is based on people’s ability to recognize, control, evaluate and change their emotions in line with their goals (Bintaş Zörer and Yorulmaz, 2022). It also has different dimensions, such as producing emotions while being regulated, regulating the processes at their source and the emotions of those around them (Gross, 1998). The functional regulation of emotions is explained by the strategies of different theorists. For example, according to Parkinson and Totterdell (1999), strategies such as trying not to think about anything or trying to think logically about the problem are included in cognitive strategies, while strategies such as acting as if you are happy and exercising are included in behavioral strategies. Additionally, some research findings produce different results in cultural context. For example, according to Wei et al. (2013), suppression of emotional expression in Eastern cultures appears to be associated with more positive outcomes in the interpersonal context. Although emotion regulation is perceived as adaptive by some and disruptive to others (Aldao et al., 2010), it offers a strategic perspective to IUMEH. For an employee who perceives happiness as an ongoing emotion rather than a momentary one and cannot concentrate, remembering his emotion regulation mechanism serves as a warning for him about the need to regain control for his ultimate goal. As a matter of fact, the feeling of happiness is experienced least frequently when the individual is alone, similar to emotions such as sadness, anger and fear. These emotions are most frequently experienced when working with a person (Scherer et al., 1988), and accordingly, it is important to activate stimulating mechanisms for the employee to achieve individual tasks. Guiding strategic maps such as this mechanism are needed, especially to regulate the emotions of employees in area C.

There is no doubt that employees like joy and happiness and are excited to do the job they love. However, permanent happiness has many disadvantages. When overjoyed, significant threats and dangers are ignored. Because the intoxication of happiness brings an invisible veil before one’s eyes while doing his/her work. His heart is filled with an eternal belief that everything will be all right. It prefers to see only the good and the beautiful, ignoring all possible consequences and risk factors. Attention deficit may occur. For example, it can be time consuming for employees to notice embedded information, especially when performing detail-oriented tasks.

In addition, it is necessary to focus on the relationship between happiness and creativity (Sugay, 2019). Despite the positive interaction between them (Fredrickson, 2004), Davis (2009) revealed that happiness can increase creativity, but the same increase is no longer witnessed when intense happiness is experienced. In terms of brain functions, when you are very happy, problem solving slows down and this is related to being less creative (Sugay, 2019).

In the light of all these explanations, IUMEH can be summarized as follows: Employee happiness makes the person motivated and productive up to a certain point. At this point, it is seen that certain stimuli accelerate the upward slope of the curve. Factors such as improving arrangements regarding the employee’s work environment, perfection in work-family-social life balance, positive thinking style and positive personality traits, and having emotion regulation and control skills are important stimuli for area A, which shows an increasing trend in IUMEH. Considering the activities that the employee has done so far in his job performance, the period when he experiences more productivity than ever is area B, which explains that IUMEH’s happiness-performance relationship is completely balanced. Area C of IUMEH is explained by the fact that an overdose of happiness delays performing activities, tends to make and carry out extraordinary (radical) decisions with momentary delusion, and ultimately results in a decrease in job performance. Decisions made while in this region may lead to consequences such as job termination and character change. In addition, differences such as culture, type and characteristics of the job, business types, generation of the manager, manager level, personality traits of the employee and type of sector should be taken into account in the interpretation of IUMEH.

## 7 Concluding remarks

Although there are many definitions of happiness in the literature, happiness in general is the sum of the satisfaction and positive emotions that individuals receive from their lives (Kangal, 2013). Although happiness is universal as an emotion, the meaning that everyone attaches to happiness is not universal, not everyone wants happiness, does not chase after happiness, and contains many cultural norms (Reiss, 1991; Gilbert et al., 2014; Muhtar, 2016; Çevik, 2020). With the definitions included in the study, the facts that happiness is personal, everyone makes sense of the concept as a result of their experiences, and the pursuit of happiness reduces the sense of personal joy have come to light. Ultimately, to make a definition, happiness is an internal reasoning that creates excitement and curiosity in people until it is felt, gives pleasure and satisfaction as soon as it is felt, and after it is felt, with the intensity of positive emotions such as joy and pleasure, it puts people in a complex emotional confusion.

Considering that the pursuit of happiness is tiring, makes us emotionally vulnerable, interferes with our private lives and makes us more lonely, −as Gruber et al. (2011) revealed- happiness is always, in all circumstances, it is necessary to rethink the expectations that it catches us in all kinds and that it will be good for the soul. The interesting thing is that chasing happiness causes people to lose their sense of “joy” (Spicer and Cederström, 2015), which can make life more meaningless. Happiness feels great when experienced, but it is momentary, temporary, and cannot exist immediately when desired. Doing his best to reach the goals and objectives, letting the events flow, living more spontaneously, keeps the excitement constantly with the feelings of curiosity and joy. The willingness to reach the basic corporate vision in order for the employees in business life to be worthy of their jobs, makes this dynamism continuous.

Measuring happiness is as difficult as measuring the warmth of the soul or finding the exact color of love (Spicer and Cederström, 2015). Moreover, although the steps taken to measure emotions and predict behaviors have improved day by day, the simpler question of “what is happiness” seems to remain unanswered. However, inferences could be presented on possible indicators that help to understand the overdose of employee happiness with IUMEH. In this way, the managers can receive individual signals regarding the organizational progress. Additionally, the measure of relationships established with employees can help them become more involved in the work, keep their performance dynamic, and accept constructive changes without high resistance. Thanks to IUMEH, a consciousness develops regarding the emotional states of the employee whose performance begins to decline. Questions such as these arise in the manager’s mind: “What are the emotional events that affect my employee?” “What are the factors that create changes in my employee’s behavior (especially hindering his performance) and what is the degree of influence of those that show positive affect among these factors?” “Although the characteristics of job do not change, which personal characteristics or socio-psychological conditions are changing? The manager searches for methods to increase his employee’s dynamism at work. In this context, techniques such as job bridging, job rotation, and job enrichment are helpful tools. A performance- and competency-based compensation system can increase the willingness of both parties to actively self-monitor and make changes if necessary, as well. Gamification methods are also beneficial in processes such as recruitment and training of employees, helping to get to know the person during the game. Thanks to the compatibility between innovative human resources practices and employee-oriented policies (Joseph et al., 2023), it becomes possible for both stakeholders to win.

Fear of happiness can be expressed as individuals preventing their own happiness with the thought that it may be a sign of pain, grief or bad events that will follow happiness. Therefore, in this study, using the “cherophobia,” which is the fear of happiness, was not found appropriate due to the fear of the happiness of others, not the person’s own. Just like happiness, the fear of happiness is also affected by the culture (Aktan and Tutar, 2007), which expresses the thought, belief and behavior patterns, experience and accumulation of the society.

Happiness cannot be measured, but it can be helped to manage stress, balance work-family life, and establish positive strong bonds rather than addictions in the work environment. For example, the Covid-19 Pandemic that broke out in 2020 showed us that the arrangements that made a difference in working hours and form had positive reflections on the productivity and motivation of the employees. With the results of the study, it seems that the flexible working hours and remote working style will maintain their popularity for a long time, and perhaps increase, despite the end of the epidemic. With the said regulations, it is possible to transform the quality of work-life and happiness into a common income for the manager and the employee. After all, the investment in people is the most valuable and the most permanent.

Although pessimistic about the phenomenon of happiness, this study reminds that an enlightened manager in the spiral of awareness-happiness-commitment should put the psychological health of his employee at the center of his strategic roadmap. It is actually beneficial for the manager to fear—or worry about–the happiness of his employee. Because when fear is experienced, the level of excitement in the person increases in order to perceive the threats developing around him and respond appropriately. Along with it, its effect on decision-making increases and the physical response to fear causes an increase in heart rate (Cacioppo et al., 1993 as cited in Gruber et al., 2011). Conversely, a lack of fear can be detrimental as it prevents you from responding appropriately when faced with a threat. When the person is not afraid, the fight-or-flight response that prompts action is delayed. Moreover, people around may not be aware that help is needed. Therefore, the manager’s fear helps to evaluate the situation, warn the employee and give an appropriate response. An overdose of happiness can turn into many negative reasons such as inefficiency, distraction, and feeling less happy. On the other hand, the employee takes his job and what he feels at work to his home. In this context, the problem of balancing work and family life is important. This situation also increases the possibility that happiness is actually a corporate social responsibility (Fisher and King, 2022).

In addition, the systematic literature review on IUMEH has some limitations. Firstly, the search sample size may be small due to the use of a single database and limited gray literature. Additionally, common titles and findings were interpreted, including those from non-open access studies, due to the desire to summarize all information in a single table. However, this study has several uses. Additionally, they attempted to create a new research agenda, which is a unique contribution to the field. For instance, the author deliberately chose a topic with a narrower scope to enhance clarity and comprehensibility. Furthermore, the article presents a shift in perspective toward the observed phenomenon, supported by available evidence. The systematic approach taken, including clear justifications for inclusion and exclusion, and a step-by-step progression, enhances the transparency and reproducibility of the study compared to traditional literature reviews. As the selected research field is still developing, it is not possible to determine the exact number of articles required (Kraus et al., 2020). Therefore, based on the implications of this study, it can be assumed that the methodology used was of high quality and the resulting final model is reliable.

Because of all these observations and research, it is clear that happiness at work and understanding its source still have contradictions at a level that will create more confusion. Nevertheless, these suggested examples of thought and research contain arguments against the thesis that employee happiness is beneficial at all times, in all cultures, to any extent. Moreover, after the pandemic, which was felt intensely between 2020 and 2022, do you think that most of the business facts that are believed to be true or real do not change? New regulations on many new issues, from working styles to working hours, from attire to new skills, affect business life and its results considerably, and the relativity of employee happiness has begun to arouse more interest. Maybe even at that time when the pandemic was intense and the bans were increasing day-by-day, happiness was the most sought-after and targeted word. Because in those days when self-centeredness increased, we kept chasing happiness.

The relationship between employee happiness and job performance is not always straightforward, as demonstrated by the ‘Inverted-U Model of Employee Happiness (IUMEH)’ and its associated outcomes. The literature on this subject has identified variations in the degree of correlation between the two factors (Hsiao et al., 2015). Simply meeting the internal needs of employees may not suffice to enhance their performance. Research and observations suggest that a two-way gain can be achieved when employees align their individual goals with those of the organization. Additionally, sustainable happiness can only be achieved by abandoning the pursuit of happiness and instead focusing on tools that help individuals feel useful and valuable throughout their lives. Over time, humans can become accustomed to the initial pleasure promised by certain things, leading to a hedonic adaptation that prevents them from experiencing the same level of pleasure repeatedly. Additionally, while happiness can vary based on circumstances and experiences, everyone experiences it at a moderate level. It is more reasonable to search for meaning and reasons that can make one’s happiness and motivation dynamic. In general, satisfaction with life or work may be related to the evaluation of the work done rather than economic or social reasons. For a low-level employee, the only reason may be to have a job. However, the expectation of being able to advance in their career in the future can serve as a motivating factor, leading to an increase in their performance day by day. As Kuhl and Atkinson (1986) and Wrzesniewski et al. (1997) argue that there is always a direct relationship between an individual’s intention and action.

In the last sentences of the article, suggestions for future research are offered. First of all, it is recommended that managers in businesses observe the job performance of their employees based on a time series and delve deeper into performance indicators with variables such as the frequency of achieving noted and clearly defined business goals in the incentive periods offered for them, organizational culture, type of job and personality traits. With the relationship analysis between variables, a graph can be created on the degree of factors affecting the final performance. The steady increase in the performance of the organization depends on the ongoing momentum in the performance of the employee, like the ring of a chain. Providing regular feedback to employees also increases their dynamism in terms of evaluating and improving their own performance. Since it is not possible to copy a talented and motivated employee, the willingness of the employee, who is considered a strategic asset for organizations, to explain his periodic performance and the reason for the changes on the graphical curve and to find solutions to current and possible problems, explains that the employee’s happiness-performance relationship is valuable for that organization. Moreover, this comes before financial matters for the employee.

In the future, it has given the insights into gender-based variations in employee happiness and delved deeper into the underlying factors contributing to these differences across cultures. Further analysis could explore societal norms, workplace dynamics, and individual experiences that shape gender-specific perceptions of happiness and its impact on job performance. Building on the findings related to sectoral differences in employee happiness and job performance, it has considered conducting a comparative analysis across industries to identify commonalities and differences in the factors influencing workplace well-being and productivity. This could involve examining sector-specific challenges, organizational practices, and cultural norms that contribute to variations in employee satisfaction and performance. In addition to quantitative analyses, it can be considered incorporating qualitative methods, such as interviews or focus groups, to capture employee perspectives on the factors influencing their happiness and performance in the workplace. Qualitative insights could provide valuable context and depth to complement quantitative findings, offering a more holistic understanding of employee experiences and perceptions. Moreover, it can be valuable to give cultural diversity highlighted in the discussion, to consider integrating cross-cultural perspectives into the analysis to explore how cultural norms and values influence the relationship between employee happiness and job performance. This could involve comparing findings across different cultural contexts to identify universal principles as well as culturally specific factors that shape workplace dynamics.

As we approach the end, a few tips can be offered to balance the happiness of employees instead of chasing happiness: increasing self-awareness, reviewing habits, finding new activities and personal development adventures, exploring social awareness, observing concentration, etc. We also know that in 2012, the United Nations declared March 20 as the International Day of Happiness in order to recognize the importance of prosperity and happiness as a universal goal for humanity. Why do not we give a holiday to those who actually work that date and support them to be happy?

There are many reasons that affect the morale and motivation of employees in business life. However, the ones that affect them the most are the managers who provide financial gain. Factors such as the level of interdependence with their managers, the commonality of goals, and the sense of worthiness have a significant impact on employee happiness. On top of that, the way managers approach situations make’s a significant difference, which is even more memorable. For example, a day in the past, while having dinner with the Turkish leader Mustafa Kemal Atatürk at a meeting, a Turkish soldier spills food on Venizelos. The soldier is crushed by an incredible shock and feels such fear! Just then, Atatürk says his historical statement: “I taught the Turkish nation everything, but I could not teach servanthood.” This approach, which was put forward in a situation where many people would be overwhelmed by stress, is astonishing. He raises a fallen man to the heights of the lofty. Finally, it is necessary to state that personal characteristics and perceptions make people happy rather than conditions. The worst enemy of happiness is the anxiety of losing it. True happiness is not to be defeated by happiness (Kaya, 2011).

## Author contributions

SK: Writing – review & editing, Writing – original draft, Visualization, Validation, Supervision, Software, Resources, Project administration, Methodology, Investigation, Funding acquisition, Formal analysis, Data curation, Conceptualization.
